# Is Gly16Arg β_2_ Receptor Polymorphism Related to Impulse Oscillometry in a Real-Life Asthma Clinic Setting?

**DOI:** 10.1007/s00408-016-9848-5

**Published:** 2016-02-15

**Authors:** Sunny Jabbal, Arvind Manoharan, Joseph Lipworth, William Anderson, Philip Short, Brian Lipworth

**Affiliations:** Scottish Centre for Respiratory Research, Ninewells Hospital and Medical School, University of Dundee, Dundee, DD1 9SY UK

**Keywords:** Asthma, Genotype, Beta-2 receptor, Impulse oscillometry

## Abstract

**Purpose:**

We evaluated whether Gly16Arg beta2-receptor genotype relates to impulse oscillometry (IOS) in a real-life clinic setting.

**Methods:**

Patients with persistent asthma taking inhaled corticosteroid ± long-acting beta-agonist (ICS ± LABA) were evaluated. We compared genotype groups comprising either no Arg copies (i.e. GlyGly) versus one or two Arg copies (i.e. ArgArg or ArgGly). IOS outcomes included total airway resistance at 5 Hz (R5), central airway resistance at 20 Hz (R20), peripheral airway resistance (R5–R20), reactance at 5 Hz, area under reactance curve (AX) and resonant frequency (RF). In addition, we recorded ACQ-5 and salbutamol use.

**Results:**

One hundred and twelve ICS-treated asthmatic patients (mean ICS dose 1238 µg/day), mean age 43 years, ACQ 2.34, FEV1 82 %, R5 177 % were identified—89 were also taking LABA. 61 patients were GlyGly, while 14 were ArgArg and 37 were ArgGly. There were no significant differences in IOS outcomes, ACQ or salbutamol use between the genotypes. The allelic risk (as odds ratio) for less well-controlled asthma (as ACQ > 1.5) was 1.1 (95 % CI 0.72–1.68) in relation to each Arg copy with a corresponding odds ratio for abnormal R5–R20 > 0.07kPA/l.s being 0.91 (95 % CI 0.57–1.44). 71 % of patients had an ACQ > 1.5 in the GlyGly group, versus 67 % in GlyArg/ArgArg group, with corresponding figures for abnormal R5–R20 > 0.07 kPa/l.s being 69 versus 73 %.

**Conclusion:**

In a real-life clinic setting for patients with poorly controlled persistent asthma taking ICS ± LABA, we found no evidence of any relationship of Gly16Arg to IOS, ACQ or salbutamol use.

## Introduction

Beta-2 adrenoceptor (β2ADR) allelic polymorphisms at position 16 (Gly16Arg) may have a clinically important effect on individual asthmatic patient’s response to beta-agonist therapy [1]. It has been shown that possession of the Arg allele results in enhanced down-regulation and uncoupling of the β2ADR with associated subsensitivity of response following exposure to regular long-acting β2-agonist (LABA) therapy in asthmatic patients receiving concurrent inhaled corticosteroids (ICS) [2]. This subsensitivity of response has been shown to result in loss of protection against bronchoconstrictor stimuli as well as worse control for exacerbations [3–5]. For example, in asthmatic children receiving ICS/LABA, each copy of the Arg allele is associated with a 1.74-fold increase in risk for asthma exacerbations [6]. Moreover, among asthmatic children with the homozygous Arg genotype, control was better when receiving ICS plus montelukast than with ICS + LABA [7].

Other studies have reported no link between Gly16Arg and pulmonary function outcomes using spirometry or airway hyper-responsiveness in adult asthmatic patients taking ICS/LABA [8–10]. However, impulse oscillometry (IOS) appears to be more sensitive than spirometry for detecting bronchodilator responses to β2-agonists in patients with asthma [11, 12]. IOS is an effort-independent technique performed during normal tidal breathing which can be used to ascertain measurements of airway resistance (R) and reactance (X), in contrast to spirometry which involves a forced expiratory manoeuvre to measure effort-dependent lung volumes and flow rates [13]. Moreover, IOS may be used to assess frequency dependence of resistance and reactance in central and peripheral airways [14].

To the best of our knowledge, no study has yet looked at the putative effect of Gly16Arg on IOS in asthmatic patients taking ICS/LABA. As a pilot study, we have therefore performed a retrospective analysis in a real-life asthma clinic setting where we performed both IOS measurements and Gly16Arg genotyping as part of routine care. In addition, we also assessed the potential impact of Gly16Arg upon asthma control using the Juniper asthma control questionnaire (ACQ) [15].

## Patients and Methods

We evaluated *n* = 112 consecutive ICS-treated non-smoking asthma patients attending our hospital clinic in order to analyse their IOS and ACQ score. Patients were divided into two genotype groups, namely those that do not possess the Arg polymorphism (*n* = 61:GlyGly) and those that possess either one or two Arg copies (*n* = 14: ArgArg or *n* = 37 ArgGly). As part of their routine workup, all patients had IOS and spirometry performed. Patient demographics and medications were recorded, as was asthma control, based on the ACQ questionnaire. Salbutamol use was recorded over the previous week. We routinely perform Gly16Arg genotyping in our clinic to help guide asthma management; hence, this audit of routine clinical care did not require internal review board ethical approval, although Caldicott guardian approval was obtained for access to the data. Lung function was measured on the morning of clinic between 9am and 11am. The patients had already taken their morning asthma medications in the usual fashion. The patients all had IOS first, followed by spirometry.

IOS (Jaeger Masterscreen IOS, Hochberg, Germany) was performed as previously described [16] in triplicate in accordance with manufacturer’s guidelines. Resistance at 5 Hz (R5) and 20 Hz (R20) is a measure of total and central airway resistance, respectively; hence, peripheral airway resistance was ascertained by the difference between R5 and R20. Reactance at 5 Hz (X5) and the area under the reactance curve (AX) were also measured. A SuperSpiro spirometer (Micro Medical Ltd, Chatham, Kent, United Kingdom) was used to perform spirometry in triplicate in accordance with European Respiratory Society guidelines [17]. Beta-2 adrenoceptor genotype was assayed using a previously validated assay [6].

## Statistical Analysis

Unpaired students *t* test or analysis of variance was used to compare normally distributed continuous variables, whereas the Mann–Whitney or Kruskal–Wallis test was used for non-normally distributed continuous variables. We compared two genotype groups comprising either no Arg copies (GlyGly) versus one or two copies (GlyArg or ArgArg). Binary logistic regression was used to calculate odds ratios for asthma that was not well controlled (i.e. ACQ > 1.5) as well as abnormal peripheral airway resistance as R5–R20 > 0.07 kPa/l.s [16], in relation to each Arg allele. Statistical significance for all analyses was set at *P* < 0.05 (2-tailed). SPSS statistical software, version 21 (SPSS Inc., Chicago, Illinois) was used for all analyses.

## Results

A total of 112 ICS-treated persistent asthmatic patients were analysed. The mean age of patients was 43 years and most of our patients (99 %) were Caucasian. Mean FEV1 was 82 % predicted, mean R5 177 % predicted, mean ACQ was 2.34, mean ICS dose was 1238 µg (beclomethasone equivalent dose), 80 % of patients were on a LABA and 36 % were on LTRA. The mean ICS dose was 1353 and 786 ug, respectively, for patients taking ICS/LABA and ICS alone (*P* < 0.0001). Genotype frequencies were as follows: GlyGly 54.5 %, ArgGly 33 %, ArgArg 12.5 %.

**Fig. 1:**
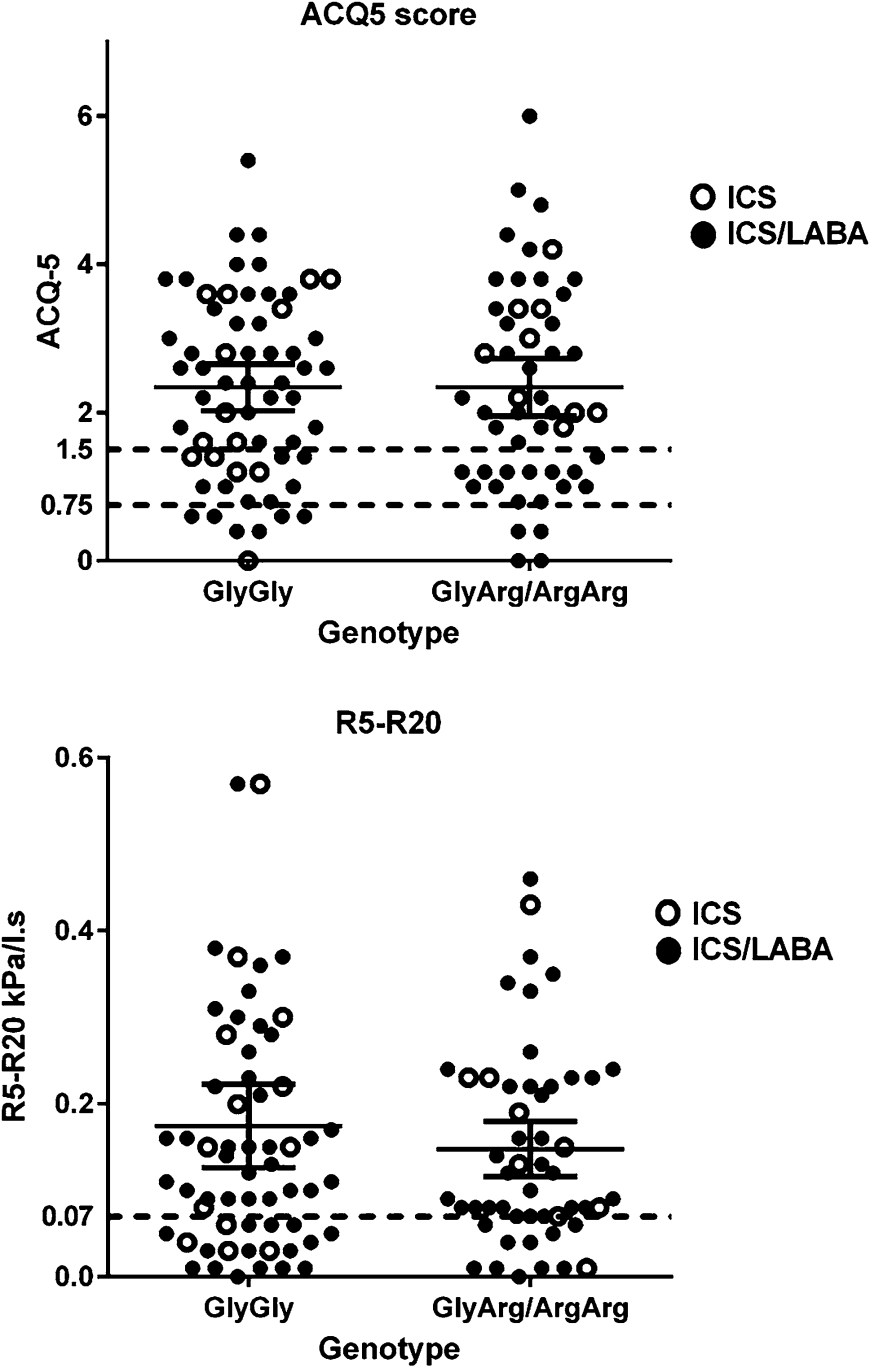
Scatter plot of individual values for ACQ-5 and R5–R20 along with means and 95 % CI for genotypes containing no Arg copies (GlyGly) and either one or two Arg copies (GlyArg/ArgArg). For ACQ, the interrupted lines show cutoffs for optimal control (<0.75) and poor control (>1.5). For R5–R20, the interrupted line depicts abnormal values >0.07 kPa/l.s. Open symbols depict ICS without LABA, while closed symbols depict ICS with LABA

Among all 112 ICS-treated patients, there was no significant difference in patient demographics between the genotype groups, aside from ICS dose. The mean ICS doses (beclomethasone equivalent) were 1136 and 1358 µg for GlyGly and ArgArg/ArgGly respectively (*P* = 0.04). Despite the lower ICS dose in the GlyGly patients, there were no significant differences in any IOS outcomes, ACQ or salbutamol use between the two groups (Table 1). We also separately compared homozygous genotypes (i.e. without the heterozygotes) which showed no differences (ArgArg vs. GlyGly): R5 0.57 versus 0.60 kPa/l.s *P* = 0.7, R20 0.41 versus 0.43 kPa/l.s *P* = 0.35, AX 1.39 versus 1.56 kPa/l *P* = 0.32, ACQ 2.87 versus 2.34 *P* = 0.16, Salbutamol use: 8.5 versus 6.5 puffs/day *P* = 0.16. The odds ratio per copy of Arg for poor asthma control (as ACQ > 1.5) was 1.1 (95 % CI 0.72–1.68) and for abnormal R5–R20 > 0.07 kPa/l.s was 0.91 (95 % CI 0.57–1.44). 71 % of patients had an ACQ > 1.5 in the GlyGly group, versus 67 % in GlyArg/ArgArg group, with corresponding figures for abnormal R5–R20 > 0.07 kPa/l.s being 69 versus 73 % (Fig. 1).

**Table 1:** Outcome comparisons between genotypes (means and 95 % CI) for all ICS-treated patients

	GlyGly (*n* = 61)	GlyArg/ArgArg (*n* = 51)	*P*
R5 (kPa/l.s)	0.60 (0.54–0.67)	0.58 (0.52–0.65)	0.69
R20 (kPa/l.s)	0.43 (0.39–0.47)	0.44 (0.39–0.48)	0.78
R5–R20 (kPa/l.s)	0.17 (0.13–0.22)	0.15 (0.12–0.18)	0.38
X5 (kPa/l)	−0.27 (−0.63–0.10)	−0.13 (−0.34–0.08)	0.38
AX (kPa/l)	1.56 (1.09–2.03)	1.31 (0.95–1.68)	0.42
RF (Hz)	17.6 (15.9–19.2)	16.66 (15.1–18.3)	0.43
ACQ5	2.34 (2.02–2.65)	2.34 (1.95–2.73)	0.99
Salbutamol (puffs/day)	6.5 (5.3–7.7)	6.41 (5.0–7.9)	0.91
FEV1(l)	2.48 (2.27–2.71)	2.40 (2.19–2.61)	0.58

When the data were filtered for LABA users (*n* = 89/112), there were also no significant differences in any outcomes when comparing ArgArg/ArgGly (*n* = 42) versus GlyGly (*n* = 47) groups: R5 0.56 versus 0.57 kPa/l.s *P* = 0.91, R20 0.42 versus 0.43 kPa/l.s *P* = 0.87, R5–R20 0.14 versus 0.15 kPa/l.s *P* = 0.16, AX 1.35 versus 1.50 kPa/l *P* = 0.45, ACQ 2.25 versus 2.37 *P* = 0.67 and salbutamol use 6.1 versus 6.5 puffs/day *P* = 0.73. However, for the R5–R20 those taking ICS/LABA had lower value compared to ICS alone: 0.15 versus 0.23 kPa/l.s *P* = 0.03. Spirometry variables were non-significant comparing ICS/LABA versus ICS alone; FEV_1_ % predicted 82 versus 80 % *P* = 0.65, FEF25-75 % predicted 51.6 versus 47.9 % *P* = 0.6. For homozygous genotypes in ICS/LABA users (ArgArg *n* = 11 vs. GlyGly *n* = 47), there were also no significant differences: R5 0.57 versus 0.60 kPa/l.s *P* = 0.70, R20 0.41 versus 0.43 kPa/l.s *P* = 0.73, AX 1.39 versus 1.56 kPa/l *P* = 0.75, ACQ 2.87 versus 2.34 *P* = 0.16 and salbutamol use 8.54 versus 6.52 puffs/day *P* = 0.16. The odds ratio per Arg copy for ACQ > 1.5 among ICS/LABA users was 1.34 (95 % CI 0.87–2.06) and for abnormal R5–20 > 0.07 kPa/l.s was 1.1 (0.70–1.75 95 % CI).

## Discussion

The results of the present study demonstrated no significant difference in IOS outcomes in relation to the Gly16Arg polymorphism in ICS-treated persistent adult asthmatics in a real-life clinic setting. Furthermore, there was also no difference in salbutamol use or asthma control between genotypes. These findings were replicated in a subgroup analysis of those patients who were taking ICS/LABA. This observation is in contrast to previous studies in asthmatic children, which have identified an increased risk of exacerbation in individuals exposed to regular ICS/LABA who possess one or more copies of the Arg allele [5, 6]. We believe that our real-world clinic patients were a strength of the study, as such patients tend to be excluded from prospective randomized controlled trials due to unrealistic inclusion and exclusion criteria. Thus, we did not select out particular phenotypes who, for example, were required to exhibit marked salbutamol reversibility, as has been the case in several previous studies where there has been a clear a prior bias towards beta-agonist responders [8, 9].

The ACQ7 score includes input for both reliever use as well as FEV_1_ % predicted. We elected to use ACQ5 for this study since concomitant LABA exposure might potentially confound ACQ scoring due to concomitant effects on reliever use as both long- and short-acting beta-agonists act on the same receptor. At the same time, we considered that including FEV_1_ % might also influence the overall ACQ score due to the bronchodilator effects of LABA. Pointedly, the ACQ-5 score has been shown to be usable in clinical trials without loss of validity compared to ACQ7 [15]. The minimal important difference in ACQ is 0.5, while the optimal cut points for well-controlled and inadequately controlled asthma are 0.75 and 1.5, respectively [18]. The mean ACQ score of 2.34 in our study exceeded the optimal cutoff of 1.5 for identifying poor asthma control [18] with 69 % of all patients having values higher than this cutoff. Despite having a relatively preserved mean FEV_1_ of 82 %, our patients exhibited a high total airway resistance (R5) of 177 % as well as a high peripheral airway resistance (R5–R20) of 0.16 kPa/l.s. This along with a mean ICS dose of 1238 µg/day with most patients taking ICS/LABA and at least 6 puffs/day of reliever use would indicate that our cohort had moderate to severe persistent asthma in keeping with a secondary care setting.

As this was a pilot study, we only captured cross-sectional data from one clinic visit, and did not assess variability over time, which is the hallmark of patients with persistent asthma. Moreover, we did not evaluate treatment compliance and so we can only assume that patients were taking what was recorded in the notes at the time of the clinic visit. We appreciate the small number of individuals possessing the homozygote Arg genotype which limited our analysis when comparing homozygous genotypes (*n* = 14 vs. *n* = 61). Nonetheless, when we analysed the data among all patients looking at the attributable risk of each Arg copy according to poor asthma control (ACQ > 1.5), we found an odds ratio of 1.1 (95 % CI 0.72–1.68) and for abnormal R5–R20 > 0.07kPA/l.s an odds ratio 0.91 (0.57–1.44).

Studies have shown corticosteroids may reverse β2ADR down-regulation and associated subsensitivity of response in asthmatics taking regular LABA [19, 20]. Considering all of our patients were taking ICS with a mean beclomethasone equivalent dose of 1238 µg/day, this may have obviated any potential differences in IOS between genotypes in ICS/LABA-treated patients. One advantage of IOS is that it can be used to evaluate the smaller airways using frequency-dependent measures such as peripheral airway resistance (R5–R20). In this regard, when inspecting the scatter plot, we identified a high proportion (71 %) of patients with abnormal R5–R20 values above 0.07 kPa/l.s, although there were no differences between genotypes.

In conclusion, our real-world pilot study demonstrated no significant impact of Gly16Arg upon IOS outcomes, salbutamol use or asthma control, in adults with persistent asthma. Further large-scale studies are required to evaluate the impact of Gly16Arg genotype on serial IOS measurements over time, perhaps comparing cohorts with and without LABA therapy.

## References

[1] Liggett SB (2000). The pharmacogenetics of beta2-adrenergic receptors: relevance to asthma. J Allergy Clin Immunol.

[2] Lipworth B (2013). beta-Adrenoceptor genotype and bronchoprotective subsensitivity with long-acting beta-agonists in asthma. Am J Respir Crit Care Med.

[3] Lee DK, Currie GP, Hall IP (2004). The arginine-16 beta2-adrenoceptor polymorphism predisposes to bronchoprotective subsensitivity in patients treated with formoterol and salmeterol. Br J Clin Pharmacol.

[4] Wechsler ME, Kunselman SJ, Chinchilli VM (2009). Effect of beta2-adrenergic receptor polymorphism on response to longacting beta2 agonist in asthma (LARGE trial): a genotype-stratified, randomised, placebo-controlled, crossover trial. Lancet.

[5] Palmer CN, Lipworth BJ, Lee S (2006). Arginine-16 beta2 adrenoceptor genotype predisposes to exacerbations in young asthmatics taking regular salmeterol. Thorax.

[6] Basu K, Palmer CN, Tavendale R (2009). Adrenergic beta(2)-receptor genotype predisposes to exacerbations in steroid-treated asthmatic patients taking frequent albuterol or salmeterol. J Allergy Clin Immunol.

[7] Lipworth BJ, Basu K, Donald HP (2013). Tailored second-line therapy in asthmatic children with the Arg(16) genotype. Clin Sci (Lond).

[8] Bleecker ER, Nelson HS, Kraft M (2010). Beta2-receptor polymorphisms in patients receiving salmeterol with or without fluticasone propionate. Am J Respir Crit Care Med.

[9] Bleecker ER, Postma DS, Lawrance RM (2007). Effect of ADRB2 polymorphisms on response to longacting beta2-agonist therapy: a pharmacogenetic analysis of two randomised studies. Lancet.

[10] Manoharan A, Anderson WJ, Lipworth BJ (2013). Influence of beta(2)-adrenergic receptor polymorphism on methacholine hyperresponsiveness in asthmatic patients. Ann Allergy Asthma Immunol.

[11] Nair A, Ward J, Lipworth BJ (2011). Comparison of bronchodilator response in patients with asthma and healthy subjects using spirometry and oscillometry. Ann Allergy Asthma Immunol.

[12] Short PM, Williamson PA, Lipworth BJ (2012). Sensitivity of impulse oscillometry and spirometry in beta-blocker induced bronchoconstriction and beta-agonist bronchodilatation in asthma. Ann Allergy Asthma Immunol.

[13] Oostveen E, MacLeod D, Lorino H (2003). The forced oscillation technique in clinical practice: methodology, recommendations and future developments. Eur Respir J.

[14] Lipworth B, Manoharan A, Anderson W (2014). Unlocking the quiet zone: the small airway asthma phenotype. Lancet Respir Med.

[15] Juniper EF, Svensson K, Mork AC (2005). Measurement properties and interpretation of three shortened versions of the asthma control questionnaire. Respir Med.

[16] Manoharan A, Anderson WJ, Lipworth J (2014). Small airway dysfunction is associated with poorer asthma control. Eur Respir J.

[17] Miller MR, Hankinson J, Brusasco V (2005). Standardisation of spirometry. Eur Respir J.

[18] Juniper EF, Bousquet J, Abetz L (2006). Identifying ‘well-controlled’ and ‘not well-controlled’ asthma using the Asthma Control Questionnaire. Respir Med.

[19] Tan KS, Grove A, McLean A (1997). Systemic corticosteroid rapidly reverses bronchodilator subsensitivity induced by formoterol in asthmatic patients. Am J Respir Crit Care Med.

[20] Aziz I, Lipworth BJ (1999). A bolus of inhaled budesonide rapidly reverses airway subsensitivity and beta 2-adrenoceptor down-regulation after regular inhaled formoterol. Chest.

